# Sex Disparity in Response to Hepatitis B Vaccine Related to the Age of Vaccination

**DOI:** 10.3390/ijerph17010327

**Published:** 2020-01-02

**Authors:** Andrea Trevisan, Alessandro Giuliani, Maria Luisa Scapellato, Simona Anticoli, Rita Carsetti, Salvatore Zaffina, Rita Brugaletta, Nicoletta Vonesch, Paola Tomao, Anna Ruggieri

**Affiliations:** 1 Department of Cardiac Thoracic Vascular Sciences and Public Health, University of Padova, 35128 Padova, Italy; marialuisa.scapellato@unipd.it; 2 Environment and Health Department, Istituto Superiore di Sanità, 00161 Rome, Italy; alessandro.giuliani@iss.it; 3 Istituto Superiore di Sanità, Center for Gender Specific Medicine, 00161 Rome, Italy; simona.anticoli@iss.it; 4 Diagnostic Immunology Unit, Department of Laboratories and B cell Pathophysiology Unit, Immunology Research Area, Bambino Gesù Children Hospital, 00165 Rome, Italy; rita.carsetti@opbg.net; 5 Occupational Medicine, Health Directorate, Bambino Gesù Children’s Hospital, 00165 Rome, Italy; salvatore.zaffina@opbg.net (S.Z.); rita.brugaletta@opbg.net (R.B.); 6 Department of Occupational and Environmental Medicine, Epidemiology and Hygiene, Italian National Workers Compensation Authority, Monte Porzio Catone, 00078 Rome, Italy; n.vonesch@inail.it (N.V.); p.tomao@inail.it (P.T.)

**Keywords:** hepatitis B, vaccine, HBs-antibodies, health care workers, sex, gender

## Abstract

Hepatitis B virus (HBV) infection is one of the major infectious hazards for health-care workers (HCWs) because of the frequency of percutaneous exposures to blood or body fluids. For this reason, all HCWs should be vaccinated, including students in medicine and health professional degree programs. The aim of this study was to assess the immune coverage to anti-HBV vaccine and long-lasting protective titres of anti-HBs antibodies in female and male students to evaluate gender-related differences in response to HBV vaccination. Data relative to anti-HBs antibody titre, sex, age, and age at vaccination were collected and analyzed from 5291 Italian students (1812 males and 3479 females) of the graduate courses at the School of Medicine, who underwent the mandatory health surveillance of workers exposed to biological risk. The results indicated that gender affects the immune response to HBV vaccine, particularly evident in the case of females vaccinated after one year of age who exhibited a statistically significant (*p* = 0.0023) 1.21-fold increase in median antibody titre with respect to males. Our findings could contribute to the optimization of HBV vaccination schedules in health surveillance of HCWs.

## 1. Introduction

Hepatitis B virus (HBV) infection is a worldwide health problem. It is estimated that there are 248 million HBV carriers in the world, of whom about 780,000 die each year due to consequences of HBV chronic infection, such as liver cirrhosis and liver cancer [1]. The implementation of vaccination programs in several countries resulted in a decreased incidence of HBV infection.

In Italy, the routine HBV vaccination program has been established since 1991, including both compulsory universal vaccination of all newborns in the first year of life and 12-year-old adolescents. This vaccination plan resulted, after almost 20 years, in a marked reduction of acute HBV incidence and disease burden, achieving the status of a low endemicity country [2].

Nevertheless, HBV infection remains a relevant cause of morbidity and mortality in the general population and an occupational risk for healthcare workers (HCWs); thus, the prevention of HBV transmission in the healthcare setting is a great concern for infection control practitioners in hospitals [3].

In Italy, HBV vaccination has been recommended for HCWs since 1985 and serologic screening for HBV markers is strongly recommended at the beginning of the occupational activity. Immunized HCWs not only protect themselves, but also prevent the spread of infection to patients and colleagues, and thus deliver safe healthcare. Despite over three decades of accumulated knowledge regarding the effectiveness and safety of the hepatitis B vaccine, there is still a sizeable proportion of HCWs in the world who never get vaccinated [3].

Recently, gender differences in immune responses to infections and vaccination, in terms of entity of responses and development of protective antibodies titre after vaccination, were hypothesized [4,5,6]. Women usually have better response to vaccination in terms of protective antibodies titre than men [7].

The present research aims to investigate the gender-related differences in the long-term response to vaccination against HBV and to examine the correlation between the anti-HBs antibody level and some variable potentially affecting the anti-HBs vaccination response, such as age at vaccination and gender.

## 2. Material and Methods

### 2.1. Study Population

Students from the Padua University School of Medicine (medicine and surgery, dentistry, and health professions) have submitted to measurement of anti-HBs antibodies since 2004 up to 2017 according to protocols defined for the health surveillance of HCWs, which are immediately before medical examination, at the beginning of the first year of courses for the students of health professions, and during the second year of courses for the students of medicine and dentistry, in correspondence with the beginning of practical training. The health surveillance started in 2004 and at first involved only students of health professions; from 2007 it was extended to students of medicine, surgery, and dentistry. The enrolment inclusion criteria included the following: (1) be born in Italy after 1 January 1980 and have adhered to the mandatory HBV vaccination according to the law 165/1991 with the recommended vaccination schedule; (2) be vaccinated at three months of age or later according to vaccination schedule, i.e., three doses (time 0, after one months, and after six months) according to the law 165/1991 and circular 20/91 only, without booster after primary cycle; (3) having an antibody titre after primary vaccination cycle ≥10 IU/L; and (4) have a vaccination certificate released by the Public Health Office.

The enrolment exclusion criteria included the following: (1) born of an HBsAg-positive carrier mother, then vaccinated at birth; or (2) had a previous HBV infection at the time of study enrolment (for this reason, 28 students were excluded and (3) were not vaccinated: 6 were HBsAg positive and 22 were anti-HBc positive (7 of which were also anti-HBe positive)).

A total of 5291 Italian students, including 1812 males (34.25%) and 3479 females (65.75%) met the enrolment inclusion/exclusion criteria. The difference between males and females attending medical school reflects the Italian data (University Board 2017, where 31.3% are males and 68.7% are females) [8].

Geographic distribution and degree course (medicine and surgery, dentistry and health professions) of the student population analyzed are reported in Tables S1 and S2 in the supplementary material.

Since the vaccine manufacturer’s recommendations suggest a paediatric dose until 15 years, we assumed that all students were vaccinated accordingly with the genetically engineered *Saccharomyces cerevisiae* yeast recombinant vaccine. Further, the time of the first dose established the age of vaccination and the interval between vaccination and antibody measurement was calculated from the third vaccine dose to the date of analysis.

Finally, to gain a better insight into potential gender differences in immune coverage related to different developmental stages of the immune system, the study group was further subdivided in two subgroups as follows: subgroup 1 (BEFORE) composed of subjects vaccinated before the first year of life (1811 students, 672 males and 1139 females, prevalently vaccinated between 60 and 120 days after birth) and subgroup 2 (AFTER) included subjects vaccinated after the first year (3480 students, 1140 males and 2340 females, prevalently vaccinated during adolescence around the twelfth year of age). The time of vaccination was ascertained from the vaccination certification provided.

### 2.2. Ethics Statement

This is an observational study in which we analyzed data obtained from a mandatory health surveillance activity on workers exposed to biological risk regulated by the national Legislative Decree 81/2008; consequently, evaluation by an ethics committee was not necessary. However, students were provided with an informational note on the processing of personal and sensitive data in which they expressed consent to the possibility that data collected may be processed anonymously for epidemiological investigations and/or for scientific research purposes.

### 2.3. Measurement of HBs Antibodies

Anti-HBs antibodies were measured by commercial Chemiluminescent Microparticle Immunoassay (CMIA) [Architect System, Abbott Ireland Diagnostics Division, Ireland, limit of detection 0–1000 IU/L, specificity 99.67% (C.I. 95% 99.22–99.89), sensitivity 97.54% (C.I. 95% 95.97–98.62)]. The measurements were performed by laboratory of clinical microbiology of Padua Hospital.

### 2.4. Statistical Methods

The primary dependent variable was the antibody titre (AbT) expressed in IU/L. Given the huge variability of the AbT values across the sampled population, some analyses were performed on the logarithm of AbT (logAbT) in order to get a more stable descriptor of immune response (even if it was less informative for individual differences). The independent variables were gender, age, age at first dose of vaccination (Age1dose), and interval, which correspond to the difference: Interval = Age − Age1dose. Age-related variables are in years.

The entity and statistical significance of the modulation exerted by gender, age, and age at first dose (age1dose) on the antibodies titre (AbT) in response to HBV vaccination was assessed by both continuous (General Linear Model Analysis of Variance) and discrete (Chi-square) approaches. The discrete approach was based upon the generation of homogeneous clusters for the AbT variable by a k-means non hierarchic procedure applied to AbT distribution over the entire data set. The mutual correlation between the measured variables was assessed by Pearson’s correlation coefficient, while linear regression analysis was applied to check for the relation between AbT and age1dose.

A derived independent variable was regime which indicated the binary categorization of subjects into two groups: BEFORE and AFTER group, according to the age of the first vaccination administration: during the first year (BEFORE) or later in life (AFTER).

All the analytical procedures were applied on the whole set of subjects and independently on the two sub-sets of subjects, the BEFORE group and the AFTER group.

The focus of the work is the characterization of gender difference in vaccination response, analyzed at different levels of definitions.

We evaluated gender difference in terms of logAbT (thus largely reducing data set variability) by parametric (analysis of variance, *t*-test) approaches and in terms of direct AbT values when we computed among the variable correlation pattern and performed cluster analysis and subsequent chi-square testing. Inferential gender comparison of both AbT and logAbT were approached by non-parametric strategies (Wilcoxon rank-test), descriptive statistics reports both continuous (mean, standard deviation), and rank-dependent (median, quartiles) indexes.

## 3. Results

The descriptive statistics for all the variables potentially affecting the antibody response to HBV vaccine (age, age at the first dose of vaccine, interval between vaccination and measurement of the AbT) at both whole set and regime-separated levels are reported in Table 1. The huge variability of the antibody titre (AbT) is mirrored by the huge standard deviation and interquartile range. As expected by the smoothing effect of logarithmic transformation on extremely high values, the log AbT variable follows a normal distribution with a substantial equivalence of mean and median values.

A sex equivalence was observed for age, interval, and age1dose, suggesting that these variables did not affect the antibody response to HBV vaccination inside each subgroup of regime: BEFORE and AFTER. However, in the AFTER subgroup of either female or male students, a 300% increase in the post-vaccination AbT titre was observed, pointing to a stronger immune response upon later vaccination compared to the early one. In addition, in the AFTER vaccination regime, a 20% increase in median values of AbT was detected in female students with respect to males. With regard to this point, it is worth noting the overwhelming effect of regime on AbT titre with a five-fold increase (AFTER/BEFORE fold change) for women and around a four-fold for men (as computed on median AbT values).

The two-way analysis of variance as applied to logAbT as a source of variation for regime and gender highlights the global trends shaping vaccination response variability (Table 2). The F-value around 1000 corresponds to an effect of regime three order of magnitudes greater than natural variability. The sex effect per se is only marginally significant (although the high number of subjects increases the power of the analysis). The significance of the sex*regime interaction term points to a different effect of sex in the two vaccination regimes, as illustrated by a 20% increase in AbT titre in the AFTER female vaccines and the lack of any sex difference in the subjects vaccinated during the first year of life (BEFORE). In the AFTER but not in the BEFORE regime, a statistically significant (*p* = 0.0023) 1.21-fold increase in median antibody titre was observed in females with respect to males. In addition (data not shown), in the AFTER but not in the BEFORE regime, males showed a significantly (*p* < 0.0001) higher number of subjects with anti-HBs levels lower than 10 IU/L compared to females.

The inferential statistics as applied separately in the two regime subgroups did not show any statistically significant difference for both AbT and logAbT variables in the BEFORE condition, but showed a marked significant difference between the two sexes in logAbT (t-value = 3.14, *p* < 0.002) and AbT (Wilcoxon test, Z = 3.01, Kruskal–Wallis Chi-square = 9.09, *p* < 0.003).

One of the issues coming from the analysis of the data is the detailed nature of the antibody titre variability at different scales which suggests the presence of discrete clusters of high and low responders. With regard to this we performed a K-means cluster analysis as applied to the AbT whole set distribution (Table 3) obtaining an optimal three-cluster partition that explains the major part of variability (94%) among the 5291 subject population.

The most populated group (low) has a median AbT titre of one order of magnitude lower than the AbT titre of both middle-high and high clusters, which prompted us to collate high and middle-high clusters in a single composite class generating a two-class partition (low/high + middle-high).

Therefore, in the subsequent contingency table, to better clarify the partition into “high and low responder subjects,” we grouped Cluster1 and Cluster2 into a single group named “high-middle,” while Cluster3 corresponded to the “low” group (Table 4). As expected, the differential distribution of males and females in the clusters was consistent with the sex disparity.

Seventy two percent of the sample belonged to the low titre, cluster3 (Table 4); this means that the variability in response to vaccination is mainly driven by the high and middle responders who showed an antibodies response to the HBV vaccination between one and two orders of magnitude higher, with respect to the population bulk.

The differential distribution of the different subject categories into the response clusters gives a confirmation of the results obtained by the continuous approach (overwhelming importance of regime, gender difference in the AFTER group) but adds an important dimension not apparent by the continuous approach: the females showed greater response to vaccination independently of the age at vaccination. In fact, in both the AFTER as well as in the BEFORE groups of vaccines, higher number of female students, with respect to males, had higher immunological responses to HBV vaccination, being in the high-middle clusters. However, only in the AFTER subgroup was the difference between sexes statistically significant (Table 4).

## 4. Discussion

HBV vaccination is strongly recommended for HCWs in several European countries and in Italy, being at high risk for exposure to blood-born infectious diseases. Although the HBV vaccine is safe and effective, the compliance among HCWs is variable in different countries in relation to different vaccination policies [9,10]. In Italy, the National Prevention Vaccination Plane 2017–2019 strongly suggests HBV vaccination in HCWs, including medical students, however a large population born after 1980 has been previously vaccinated.

Immunity after HBV vaccination, although long lasting, declines over time [11]. Limited data are available about the level of protective post-vaccination anti-HBs antibodies in relation to the age of vaccination (childhood or puberty and adulthood). To render more complicated the evaluation of the durability of vaccine efficacy, a sex difference in immune responses to vaccination, in terms of entity of the immune responses to infections and vaccination, has been recently reported. Gender medicine has been applied to the occupational medicine as a new approach for the risk evaluation and management in workplaces. European roles and local laws are spreading to create the knowledge and awareness of the influence of sex/gender among all health professionals and to implement policies to address gender in health systems.

Similar to the more intense humoral and cellular immune responses to the infections, sex disparity in response to some vaccines, such as influenza, hepatitis B, tetanus, diphtheria, and yellow fever, among others, has been reported [12]. As a paradigmatic case, women usually develop a higher titre of neutralizing antibodies and a better subsequent response to the influenza vaccination [13]; however, side effects have been reported more frequently in females than in males [5,6,14]. Moreover, women vaccinated with a half dose of the seasonal influenza vaccine have been shown to mount antibody response levels similar to men vaccinated with the entire dose [15], suggesting that gender personalized vaccinations could be adequate, at least in some cases.

Gender disaggregated data of vaccine-induced anti-HBs antibodies are not available for the general population and HCWs. In order to evaluate gender-related differences, the present study analyzed the data relative to students attending to the Padua University School of Medicine. The examination of AbT in relation to different parameters potentially affecting the anti-HBs titre in response to vaccination, such as gender, age at the first dose of vaccination, and interval since the last dose of vaccine, revealed a close relation when the statistical analysis was applied. This study confirmed our previous results demonstrating that vaccination after one year of age induced greater and longer lasting protective immune responses than vaccination at three months of age [16], thus indicating that higher probability to find protective anti-HBs levels several years after vaccination is associated with age at immunization, or otherwise with less probability of disappearance of circulating antibodies. These results are consistent with other studies reported in the literature [17].

To the best of our knowledge, this is the first study that demonstrates a significant gender-related difference in terms of a higher female response. This effect was particularly evident in the case of late vaccination: females showed 20% higher vaccination-related AbT than males. The huge presence of two neatly separated groups of subjects corresponding to about three months of age and twelve years at the time of vaccination, respectively, generated a natural partition into BEFORE (vaccination at <1 year of age) and AFTER classes as the age at first dose of vaccination. This neat partition reflected on the “interval” variable, reporting the time interval from vaccination to response measurement. As a matter of fact, it has been recently demonstrated that the vaccine-induced AbT depends on the regime (that is, the time of vaccination, BEFORE or AFTER one year of age) and not on the interval between vaccination and measurement of AbT [16]. Regime and sex significantly influenced antibody response only in those vaccinated AFTER one year of age. At the moment, the only plausible explanation of this evidence could be that if innate immunity is more or less the same in both sexes, adaptive immunity is more pronounced in females [12].

In individuals vaccinated earlier than one year of age, we could report that sex didn’t affect the AbTs, whereas the age at which the vaccination was received positively influenced the efficacy of vaccination in terms of long lasting protective titre of anti-HBs antibodies, mainly evident for female subjects. This places the attention on a sex-disaggregated evaluation of the efficacy data during the vaccine clinical trials and on a sex-personalized vaccination schedule.

In general, the high variability of the antibody responses let us identify three subgroups of individuals with high, medium, and low levels of vaccine-related antibodies titre.

Several studies reported the immune privilege of females with respect to males, in murine animal models as well as in humans [5,18,19], and in response to different vaccines [12]. Usually, protective antibodies to several vaccinations are more elevated in females compared to males at all ages, as in the case of vaccination against single-stranded RNA viruses, such as trivalent seasonal influenza vaccine, measles, mumps, yellow fever, dengue, hepatitis A and B, and rubella, but also against DNA viruses such as HBV and herpes simplex vaccinations. Innate and adaptive immune responses to vaccination exhibit relevant gender-related differences [12], and females subjects show a greater humoral and cell-mediated immune response [20]. Genes for the toll-like receptor pathway and type I interferon induction justifies these differences [21] because several genes that are immune-related are located on the X chromosome and play a pivotal role in immune competence [22]. The scant available knowledge of the molecular mechanisms involved suggest sex differences in pathogen recognition capabilities and molecules, such as the Toll-like receptor 7 molecule that is encoded on the X chromosome. In addition, after immunization women have higher levels of CD4+ lymphocytes and production of Th1 cytokines, which can account for the more prompt and intense response to antimicrobial vaccination [5,7].

## 5. Conclusions

The present study is the first research disaggregating data by sex for the analysis of the immune response to HBV vaccination in medical students. Our data analysis consistently reports greater immune reactivity of women than men, as more female students were in the high responders, whereas male students were mostly included in the low responders. In the correlation analysis it was evident that sex differences in response to the anti-HBV vaccine were in the late vaccination regime, that is, in those individuals receiving the vaccine after one year of age. The discovery of meaningful association between factors modulating the HBV vaccine-antibody responses could help optimize the HBV vaccination schedule and improve the health surveillance protocols in HCWs, as well as in the general population. Further, as recently demonstrated [23], the female sex has a lower number of subjects with an antibody level lower than 10 IU/L and a faster and more intense (about twice) antibody response.

## Figures and Tables

**Table 1:** Descriptive statistics of the whole data set either aggregated and subdivided by gender (upper part of the table) and the descriptive statistics distinguished by regime (BEFORE/AFTER corresponds to the threshold of one year of age at vaccination) (bottom part of the table). It is important to note how this binary classification of regime stems directly from the data set, being the first and third quartile (Q1, Q3) of age at first dose equal to 0.23–0.27 and 11.17–11.82 for BEFORE and AFTER classes (females, males have very similar values), respectively, thus pointing to the existence of two separated classes of infant and adolescent age vaccination regimes.

**Whole Set**															
**All Subjects (n = 5291)**						**Females (n = 3479)**					**Males (n = 1812)**				
**Variable**	**Mean**	**Median**	**SD**	**Q1**	**Q3**	**Mean**	**Median**	**SD**	**Q1**	**Q3**	**Mean**	**Median**	**SD**	**Q1**	**Q3**
**age** **(years)**	21.97	21.27	2.3	20.24	23.22	21.79	21.05	2.29	20.01	23.14	22.31	21.65	2.31	20.72	23.44
**age1dose** **(years)**	7.33	11.17	5.38	0.27	11.62	7.51	11.17	5.33	0.28	11.62	6.98	11.14	5.46	0.27	11.58
**interval (years)**	13.98	12.86	4.88	9.49	19.13	13.63	12.39	4.93	8,9	18,89	14.65	13.63	4.72	10.57	19.55
**anti-HBs** **(IU/L)**	246.44	88	320.66	30	315	255,64	94	326	32	335	228.77	78	309.46	28	274.5
**logAB**	4.61	4.48	1.4	3.4	5.75	4.66	4.54	1.4	3.46	5.81	4.52	4.36	1.39	3.33	5.62
**Regime**															
**After**								**Before**							
**Variable**	**Mean**	**Median**	**SD**	**Q1**	**Q3**					**Variable**	**Mean**	**Median**	**SD**	**Q1**	**Q3**
**age** **(years)**	22.36	21.96	2.5	20.14	24.11	**Females** **(n = 2340)**		**Females** **(n = 1139)**		**age** **(years)**	20.63	20.54	1.02	19.82	21.16
**age1dose** **(years)**	11.04	11.44	2.07	11.17	11.8					**age1dose** **(years)**	0.27	0.25	0.09	0.23	0.27
**interval (years)**	10.72	10.27	3.12	8.17	12.46					**interval (years)**	19.6	19.53	1.03	18.79	20.16
**anti-HBs** **(IU/L)**	335.14	171	354.59	53.5	526.5					**anti-HBs IU/L**	92.31	34	163.33	19	85
**logAB**	5.08	5.14	1.34	3.98	6.26					**logAB**	3.78	3.52	1.09	2.94	4.44
**age** **(years)**	23.16	22.9	2.45	21.32	24.53	**Males** **(n = 1140)**		**Males** **(n = 672)**		**age** **(years)**	20.88	20.84	0.95	20.3	21.36
**age1dose** **(years)**	10.94	11.42	2.29	11.17	11.82					**age1dose** **(years)**	0.27	0.25	0.11	0.23	0.28
**interval (years)**	11.6	11.03	3.12	9.18	13.09					**interval (years)**	19.82	19.81	0.97	19.24	20.34
**anti-HBs** **(IU/L)**	307.45	141.5	347.2	43.5	460.5					**anti-HBs IU/L**	95.23	37	157.7	18	89.5
**logAB**	4.93	4.95	1.38	3.77	6.13					**logAB**	3.83	3.61	1.1	2.89	4.49

**Table 2:** Analysis of variance (general linear model) applied to the logarithm of antibody titre on whole set. The shift to the logarithm allowed for smoothing the outlier values, with subsequent normal distribution. Of note is the statistical significance of the sex*regime interaction term, whose F-value of 6.99 is greater than 4.85 for the sex effect (see text results).

	F-Values	*p*
sex	4.85	0.0276
regime	1123.47	<0.0001
sex*regime	6.99	0.0082

**Table 3:** K-means cluster analysis procedure, as applied to the whole data set, generated an optimal three-cluster partition explaining the major part of variability (94%) among the 5291 subject population. We could identify the presence of clear discrete “vaccination response” groups.

Cluster	N of Subjects	Antibody Titre (Median AbT IU/L)	Q1 (AbT)	Q3 (AbT)
high	735	960	910	1000
middle-high	766	401	319	512
low	3790	47	22	105

**Table 4:** Contingency table and relative inferential statistics for the regime and GENDER correlation (distribution across response clusters). The differential distribution of the different subject categories into the response clusters confirms the analysis of variance approach (overwhelming importance of regime, gender difference reaching statistical significance in the AFTER group). The percentages are inserted in parentheses.

**(a) Regime Effect**			
Regime	High/Middle-High	Low	Chi-Square
AFTER	1352 (38.9)	2128 (61.1)	549.7 *
BEFORE	149 (8.2)	1662 (91.8)	
**(b) Gender Effect on the Whole Set and Regime**			
	High/Middle-high	Low	Chi-Square
ALL			8.38 **
females	1032 (29.7)	2447 (70.3)	
males	469 (25.9)	1343 (74.1)	
BEFORE			0.09 ***
females	92 (8.1)	1047 (91.9)	
males	57 (8.5)	615 (91.5)	
AFTER			
females	940 (40.2)	1400 (59.8)	5.24 ****
males	412 (36.1)	728 (63.9)	

Legend: * *p* <0.0001 ** *p* = 0.0042, *** *p* = 0.76, **** *p* = 0.024.

## Supplementary Materials

The following are available online at https://www.mdpi.com/1660-4601/17/1/327/s1, Table S1: Geographic distribution of Medical School Students of Padua University, Table S2: Distribution of Medical School Students of Padua University according to degree course.
