# Supplementary material for: Sex Disparity in Response to Hepatitis B Vaccine Related to the Age of Vaccination
Source: Int J Environ Res Public Health. 2020 Jan 2;17(1):327. doi: 10.3390/ijerph17010327 (PMC6981715; doi:10.3390/ijerph17010327)
Supplement: Supplementary file 1 [file ijerph-17-00327-s001.pdf]

Table S1.

Geographic distribution of Medical School Students of Padua University

			Northern Italy		Central Italy		Southern Italy	
		**N**	**N**	**%**	**N**	**%**	**N**	**%**
**AFTER subgroup**	**All subjects**	4068	3898	95.8	38	0.9	132	3.2
	**Males**	1394	1333	95.6	16	1.1	45	3.2
	**Females**	2674	2565	95.9	22	0.8	87	3.3
**BEFORE subgroup**	**All subjects**	3814	3511	92.1	85	2.2	218	5.7
	**Males**	1388	1249	90.0	38	2.7	101	7.3
	**Females**	2426	2262	93.2	47	1.9	117	4.8

Table S2.

Distribution of Medical School Students of Padua University according to degree course

			Medicine		Dentistry		Health Professions	
		**N**	**N**	**%**	**N**	**%**	**N**	**%**
**AFTER subgroup**	**All subjects**	4068	2009	49.4	180	4.4	1879	46.2
	**Males**	1394	811	58.2	99	7.1	484	34.7
	**Females**	2674	1196	44.7	81	3.0	1397	52.2
**BEFORE subgroup**	**All subjects**	3814	1763	46.2	78	2.0	1973	51.7
	**Males**	1388	848	61.1	45	3.2	495	35.7
	**Females**	2426	915	37.7	32	1.3	1479	61.0
